# Supplementary material for: Genome-Wide Identification of Catalase Gene Family and the Function of SmCAT4 in Eggplant Response to Salt Stress
Source: Int J Mol Sci. 2023 Nov 30;24(23):16979. doi: 10.3390/ijms242316979 (PMC10706941; doi:10.3390/ijms242316979)
Supplement: Supplementary file 1 [file ijms-24-16979-s001.zip › ijms-2722993-supplementary.pdf]

International Journal of
Molecular Sciences

MDPI

Supplementary Materials

Table S1. Sequences of primer pairs were used in this study.

Primer Name	Forward (5′ to 3′)	Reserves (5′ to 3′)	
SmCAT1	CTCTTACAGTTGCCATCGGACAAGG	GGGTCAAGTGAGCGTTAGCATAGTC	RT-qPCR
SmCAT2	TCTCAGACTCTTGGAGCCCATTAGG	CTGCCAGGGTGAAAGGGAACATC	RT-qPCR
SmCAT3	AGGTCACTGGTGGTCCGACTATTG	CATCTGGTAACCGCCCTTCCTTTG	RT-qPCR
SmCAT4	GCTTGCTGGAGTTGTTGCTGTTG	CGCCCTTCTTCTGGTGAGTTTGATG	RT-qPCR
SmActin	CACTTAGCACCTTCCAGCAGATGT	GTACAACAGCAGACCTGAGTTCACT	RT-qPCR
SmTAS14	GGACAGCAGGAAGGTGGATA	TCATTCCAGGGATCTTGTCC	RT-qPCR
SmDHN1	CAACTCATGCTGCACAAACA	CAATTCATGCCAGGGATCTT	RT-qPCR
SmCAT1-<br>pBinGFP2	CTGTACAAGGGTACCCCCGGGATG<br>GATCTCTCTAAGTATCGCCCT	AGAGGATCCGTCGACCCCGGGT<br>CACATTGTAGGCTTGACAGTGAGA	Subcellular<br>localization
SmCAT2-<br>pBinGFP2	CTGTACAAGGGTACCCCCGGGATG<br>GATCCTTACAAGTACCGTCCG	AGAGGATCCGTCGACCCCGGGTCAT<br>ATGCTTGGTCTCACATTAAGC	Subcellular<br>localization
SmCAT3-<br>pBinGFP2	CTGTACAAGGGTACCCCCGGGAT<br>GGATCCTTACAAGTATCGCCC	AGAGGATCCGTCGACCCCGGGTCAC<br>ATTGTAGGCCTTACATTGAGA	Subcellular<br>localization
SmCAT4-<br>pBinGFP2	CTGTACAAGGGTACCCCCGGGATG<br>CAGTATCGCCCTTCAAGC	AGAGGATCCGTCGACCCCGGGTCAC<br>ATTATAGCAGGCTTCATAGTGA	Subcellular<br>localization
SmCAT4-GST	GGGATCCCCGGAATTCCCGGGATG<br>CAGTATCGCCCTTCAAGC	GGCCGCTCGAGTCGACCCGGGTCACA<br>TTATAGCAGGCTTCATAGTGA	Prokaryotic<br>expression
SmCAT4-<br>VIGS	GGGGACAAGTTTGTACAAAAAAGCA<br>GGCTTCAGATGAGAATGGTTGGAATG	GGGGACCACTTTGTACAAGAAAGCTGG<br>GTCGTTTCAAGATCAAAGCTAAA	VIGS
